# Implementation of a new histological grading system in ovarian mucinous carcinomas and its association with the risk of recurrence: a retrospective cohort study

**DOI:** 10.1590/1516-3180.2025.3034.12112025

**Published:** 2026-02-02

**Authors:** Adriana Yoshida, Liliana Aparecida Lucci De Angelo Andrade, Ricardo Ruiz Garcia de Almeida, Helymar da Costa Machado, Luís Otávio Sarian, Sophie Derchain

**Affiliations:** I Physician, Departamento de Obstetrícia e Ginecologia, Faculdade de Ciências Médicas, Universidade Estadual de Campinas (Unicamp), Campinas (SP), Brazil.; II Professor, Departamento de Patologia, Faculdade de Ciências Médicas, Universidade Estadual de Campinas (Unicamp), Campinas (SP), Brazil.; III Undergraduate Student, Departamento de Obstetrícia e Ginecologia, Faculdade de Ciências Médicas, Universidade Estadual de Campinas (Unicamp), Campinas (SP), Brazil.; IV Statistician, Departamento de Obstetrícia e Ginecologia, Faculdade de Ciências Médicas, Universidade Estadual de Campinas (Unicamp), Campinas (SP), Brazil.; V Professor, Departamento de Obstetrícia e Ginecologia, Faculdade de Ciências Médicas, Universidade Estadual de Campinas (Unicamp), Campinas (SP), Brazil.; VI Professor, Departamento de Obstetrícia e Ginecologia, Faculdade de Ciências Médicas, Universidade Estadual de Campinas (Unicamp), Campinas (SP), Brazil.

**Keywords:** Ovarian neoplasms, Cystadenocarcinoma, Adenocarcinoma, mucinous, Neoplasm grading, Prognosis, Ovarian mucinous carcinoma, Grade, Grading, Infiltrative growth, Prognostic

## Abstract

**BACKGROUND:**

This retrospective cohort study evaluated the prognostic significance of the GrowthBased Grade (GBG) system compared to International Federation of Gynecology and Obstetrics (FIGO) grading in ovarian mucinous carcinoma (OMC). Although FIGO grading is commonly used, its prognostic value remains controversial. The GBG system, which classifies tumors as low-grade (G1) or high-grade (G2) based on the proportion of infiltrative growth, has emerged as a potential prognostic tool.

**OBJECTIVES:**

To assess the prognostic significance of GBG and compare it with FIGO grading in OMC.

**DESIGN AND SETTING:**

This retrospective cohort study included 37 women with OMC treated at a single institution between 2009 and 2022.

**METHODS:**

GBG was determined by a histopathological review of hematoxylin and eosin-stained slides. Clinical and demographic data, including FIGO stage, CA125 levels, surgical procedures, and follow-up information, were collected. Kaplan-Meier analysis and Cox regression were used to assess the associations between GBG grading, FIGO stage, and survival outcomes.

**RESULTS:**

GBG 2 tumors were significantly associated with elevated CA125 levels, advanced FIGO stage (III), and bilaterality. Multivariate analysis showed that GBG 2 conferred a 5.4-fold higher risk of recurrence compared with GBG 1. While FIGO stage III was predictive of overall survival, FIGO grading was not associated with recurrence risk.

**CONCLUSION:**

This study suggests a potential prognostic value of the GBG system in mucinous ovarian carcinoma. GBG 2 tumors showed a higher risk of recurrence than GBG 1 tumors, whereas FIGO grading showed no such association. These findings align with previous reports and should be interpreted in the context of additional studies to clarify the system’s clinical relevance.

## INTRODUCTION

 Primary mucinous carcinoma of the ovary is rare, accounting for only 3% of all ovarian carcinomas.^1,2^ Historically, ovarian mucinous carcinoma was diagnosed in 15% of ovarian carcinoma cases; however, many of these were later identified as metastatic tumors, resulting in significant overdiagnosis. The therapeutic approach remains challenging, with ongoing debates on multiple aspects. These include the role of systematic lymphadenectomy, the necessity of adjuvant chemotherapy in cases of tumor rupture, and the optimal choice of chemotherapeutic agents: carboplatin and paclitaxel versus capecitabine and oxaliplatin.^3^

 Approximately 80% of cases are diagnosed at stage I according to the International Federation of Gynecology and Obstetrics (FIGO) classification,^1,4^ with a five-year survival rate of 83% in women with the disease confined to the ovaries. In contrast, patients with advanced disease (stages III and IV) have a five-year survival rate of only 14%.^5^

 Differentiating ovarian mucinous carcinoma from ovarian metastases requires a combination of clinicopathological evaluations, imaging studies, and immunohistochemical analyses. Most ovarian metastases originate from gastrointestinal tract cancers, but can also be secondary to breast, cervical, or endometrial cancers.^4^ Serum tumor markers such as carcinoembryonic antigen (CEA), cancer antigen 19-9 (CA19-9), and the CA125/CEA ratio,^6^ along with imaging studies — including total chest and abdominal computed tomography (CT), total abdominal magnetic resonance imaging (MRI), upper gastrointestinal endoscopy, and colonoscopy — can assist in the diagnostic process.^4^

 The following findings suggest a primary ovarian tumor: a tumor larger than 10 cm, unilateral involvement, absence of mucin in the peritoneal cavity, and a normal appendix. Histological diagnosis of ovarian mucinous carcinoma requires evidence of complex malignant cell proliferation covering an area greater than 10 mm^2^ in histological sections of the tumor.^4^ Adequate tumor sampling — collecting one to two tissue samples per centimeter of the tumor’s largest diameter — and a thorough review by an experienced pathologist are essential. Immunohistochemical profiling of ovarian mucinous carcinomas primarily relies on markers such as CK7, CK20, and CDX2 to differentiate them from primary gastrointestinal tract carcinomas.^4^ However, diagnostic challenges persist due to frequent overlaps in immunohistochemical staining patterns, necessitating correlation with imaging studies.^7^ In some cases, ovarian mucinous carcinoma remains a diagnosis of exclusion when extensive investigations fail to identify disease at another site.^8^

 Although there is no definitive evidence regarding the prognostic role of histological grading in ovarian mucinous carcinomas, the International Collaboration on Cancer Reporting recommends using the endometrioid carcinoma grading system if grading is performed.^9^ This corresponds to the FIGO grading system, which classifies tumors as well-differentiated (G1), moderately differentiated (G2), or poorly differentiated (G3) based on the proportion of solid glandular components and the presence of nuclear atypia.^9^ In 2014, the World Health Organization (WHO) officially recognized a classification previously described by Lee and Scully in 2000,^10^ which categorizes ovarian mucinous carcinomas into expansile and infiltrative subtypes based on stromal invasion patterns. The expansile subtype exhibits a confluent glandular growth pattern with minimal or no destructive stromal invasion, whereas the infiltrative subtype is characterized by overt destructive stromal invasion by nests of cells, glands, or isolated tumor cells, often associated with a desmoplastic stromal reaction.^10^

 In 2020, Busca et al.^11^ proposed a novel grading system for ovarian mucinous carcinomas, the Growth-Based Grade (GBG), classifying tumors as low-grade (G1) or high-grade (G2): G1 when the growth pattern is only expansile or infiltrative in ≤ 10% of the tumor and G2 when infiltrative invasion exceeds 10%. In their study, staging, GBG, and Silverberg histological grading^12^ were associated with disease-free survival.^11^ A subsequent validation study by the same research group confirmed that GBG G2 tumors had a higher recurrence risk than GBG G1 tumors. The Silverberg and FIGO classification systems also showed a correlation with progression-free survival in univariate analysis. However, multivariate analysis indicated that only GBG was statistically significant. In addition, the percentage of infiltrative growth was identified as the sole predictive factor for disease-specific survival.^13^

 The prognostic role of histological grading in ovarian mucinous carcinomas appears to be less significant than the invasion type classified by the GBG system,^11^ which is endorsed in the latest edition of the WHO Classification of Tumors — Tumors of the Female Genital Tract.^14^ However, the same edition acknowledges that no definitive consensus has been reached regarding which grading system should be used, as the FIGO histological grading system may also provide useful prognostic information to guide appropriate management strategies. 

## OBJECTIVE

 This study aimed to assess the prognostic significance of GBG histological grading and compare it with FIGO grading in a cohort of 37 women with primary ovarian mucinous carcinoma. 

## METHODS

### Study design and population

 This retrospective study was based on a convenience sample, and was approved by the Research Ethics Committee of Unicamp (approval number 1092/2009 and CAAE: 33451720.7.0000.5404). Women referred for adnexal masses to the Ovarian Oncology outpatient clinic at the Women’s Hospital, Prof. Dr. José Aristodemo Pinotti, CAISM-Unicamp, were selected. All participants signed an informed consent form during their first outpatient visit between December 2009 and July 2022 (N = 1,950). Patients histologically diagnosed with primary ovarian mucinous carcinoma were included in this study (n = 45). 

 Seven women were excluded because of the unavailability of hematoxylin and eosin (H&E)-stained slides for review by a gynecologic pathologist (L.A.L.A.A), and one was excluded because of a diagnosis of teratoma with a 1 mm mucinous adenocarcinoma focus, resulting in a final cohort of 37 women. Of these patients, 36 were diagnosed surgically. One patient was diagnosed via percutaneous core needle biopsy, which was performed due to the suspicion of an adnexal mass based on physical examination, serum tumor marker assessment, and imaging studies. 

### Data collection and definitions

 This study did not alter the standard hospital treatment protocols for women with ovarian mucinous carcinoma. Immunohistochemical staining was performed on tumor samples from 33 women to aid in the differential diagnosis and characterization of the tumor subtypes. All H&E-stained slides were reviewed by a single pathologist with extensive expertise in gynecologic pathology and classified strictly according to the diagnostic criteria proposed by Busca et al.,^11^ for study purposes only and without any clinical application. Following treatment, patients were followed up in outpatient clinics, and their status was updated through electronic medical records until November 2024. A subset of women who were lost to follow-up or discharged from the hospital was contacted by phone, while the survival status of the remaining patients was confirmed through two national databases: the CPF (Brazilian national identification number, canceled upon death) and the National Registry of Deceased Persons. 

### Statistical analysis

 To compare categorical variables between groups (G1 and G2 of the GBG), the chi-square test or Fisher’s exact test was used, as appropriate. Numerical variables were compared using the Mann–Whitney U test for two-group comparisons and the Kruskal-Wallis test for three-group comparisons. Disease-free survival and overall survival curves were analyzed using the Kaplan–Meier method, and comparisons were performed using the log-rank test. Factors associated with survival outcomes were assessed using both univariate and multivariate Cox regression analyses, employing a stepwise selection criterion for variable inclusion. The significance level was set at 5% (P < 0.05). Statistical analyses were performed using SAS for Windows (Statistical Analysis System), version 9.4 (SAS Institute Inc., 2002–2012, Cary, NC, North Carolina). 

## RESULTS

**Table 1** presents the demographic and clinical characteristics of the two groups of women classified according to the GBG grading of ovarian mucinous carcinomas. GBG 2 tumors were associated with elevated CA125 levels, advanced stage (III), and bilaterality compared with GBG 1 tumors. The sample predominantly included white participants with a smaller proportion of women of other ethnicities. Owing to the limited sample size, racial diversity was restricted. 

**Table 1:** Demographic, clinical, and pathological characteristics of women according to the growth-based grading (GBG) classification of tumors

		**Growth-Based Grading**		
		**GBG 1**	**GBG 2**	**P**
Age n (%), years				
	< 50	10 (37)	4 (40)	1
	≥ 50	17 (63)	6 (60)	
BMI n (%), kg/m^2^				
	< 30	19 (70)	5 (50)	0
	≥ 30	8 (30)	5 (50)	
Race* n (%)				
	White	14 (70)	6 (86)	0.633
	Non-white	6 (30)	1 (14)	
Menopausal status n (%)				
	Premenopausal	11 (40)	2 (20)	0.44
	Postmenopausal	16 (60)	8 (80)	
CA125 median, U/mL		60.26	253.95	**0.014**
CEA median, ng/mL		3.35	3.38	0.62
FIGO Stage n (%)				
	I and II	26 (96)	5 (50)	**0.003**
	III	1 (4)	5 (50)	
Tumor size median, cm†		25	21.5	0.421
Laterality n (%)				
	Bilateral	0 (0)	3 (30)	**0.015**
	Unilateral	27 (100)	7 (70)	
Lymphadenectomy‡ n (%)				
	No	12 (44)	6 (60)	0.476
	Yes	15 (56)	4 (40)	
Cyst rupture n (%)				
	No	18 (67)	4 (40)	0.258
	Yes	9 (33)	6 (60)	
FIGO Grade n (%)				
	Well differentiated	18 (67)	3 (30)	0.067
	Moderately differentiated	9 (33)	7 (70)	
Chemotherapy n (%)				
	No	20 (74)	4 (40)	0.118
	Yes	7 (26)	6 (60)	
Recurrence n (%)				
	No	26 (96)	8 (80)	0.172
	Yes	1 (4)	2 (20)	
Death n (%)				
	No	23 (85)	6 (60)	0.174
	Yes	4 (15)	4 (40)	
Final Status n (%)				
	Death	4 (15)	4 (40)	0.171
	Alive	1 (4)	1 (10)	
	Alive without disease	22 (81)	5 (50)	

BMI, body mass index

* Data unavailable for 10 women

† n = 36, as one case was diagnosed via percutaneous biopsy

‡ Pelvic and para-aortic lymphadenectomy

FIGO, International Federation of Gynecology and Obstetrics

 Regarding surgical treatment, 28 women (75.7%) underwent omentectomy, and 23 (62.2%) underwent appendectomy. Uterine and contralateral ovary preservation was performed in six (16.2%) of the 37 women. Thirty women (81.1%) were diagnosed at stage I, one (2.7%) at stage II, and six (16.2%) at stage III. The tumors were classified as GBG 1 in 27 women (73%) and GBG 2 in 10 women (27%). 

**Tables 2** **and** **3** present the results of univariate and multivariate Cox regression analyses of factors related to disease-free survival. Multivariate analysis indicated that GBG grading significantly influenced the recurrence risk, with GBG 2 associated with a 5.4-fold increased risk of recurrence compared to GBG 1. 

**Table 2:** Results of univariate Cox regression analysis for disease-free survival (n = 37)

**Variable**	**Categories**	**P Value**	H.R.*	**95% CI**
Age	Continuous variable (years)	0.979	0.999	0.936–1.066
BMI	Continuous variable	0.606	1.043	0.888–1.225
FIGO Grade	Well-differentiated (ref.)	–	1	–
	Moderately differentiated	0.631	1.82	0.16–20.84
GBG	G1 (ref.)	–	1	–
	G2	0.011	5.4	1.01–53.23
Complete Staging	No (ref.)	–	1	–
	Yes	0.839	0.75	0.05–11.99
Cyst Rupture	No (ref.)	–	1	–
	Yes	0.612	0.52	0.04–6.34
FIGO Stage	I–II (ref.)	–	1	–
	III	0.141	8.08	0.5–130.16

* HR (Hazard Ratio), risk ratio for recurrence (n = 34 censored, n = 3 recurrences)

CI, confidence interval; BMI, body mass index. Ref., reference category; FIGO, International Federation of Gynecology and Obstetrics

**Table 3:** Results of multivariate Cox regression analysis for disease-free survival (n = 37)

**Variable**	**Categories**	**P Value**	H.R.*	**95% CI**
GBG	G1 (ref.)	–	1	–
	G2	0.011	5.4	1.01–53.23

* HR (Hazard Ratio), risk ratio for recurrence (n = 34 censored, n = 3 recurrences)

CI, confidence interval; Ref, reference category

**Figure 1:**
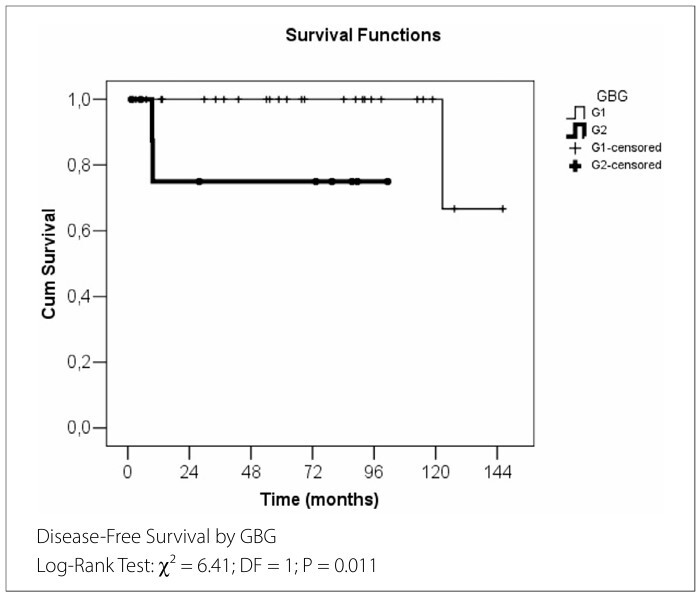
Disease-Free Survival analysis for significant variables (in months).

 Three patients experienced recurrence (one in the GBG 1 group and two in the GBG 2 group), with a mean disease-free survival time of 130.3 months in the entire cohort. **Figure 1** presents the Kaplan-Meier disease-free survival analysis for the significant variables identified in **Tables 2** **and** **3**. 

**Tables 4** **and** **5** present the results of the univariate and multivariate Cox regression analyses of factors associated with overall survival. In the multivariate model, FIGO stage III remained a significant predictor of mortality, with a 15.2-fold higher risk of death compared with earlier stages. 

**Table 4:** Results of the univariate Cox regression analysis for overall survival (n = 37)

**Variable**	**Categories**	**P Value**	H.R.*	**95% CI**
Age	Continuous variable (years)	0.067	1.049	0.997–1.103
BMI	Continuous Variable (years)	0.608	1.028	0.925–1.143
FIGO Grade	Well-differentiated (ref.)	–	1	–
	Moderately differentiated	0.628	0.7	0.17–2.94
GBG	G1 (ref.)	–	1	–
	G2	0.025	7.19	1.29–40.13
Complete Staging	No (ref.)	–	1	–
	Yes	0.146	0.3	0.06–1.53
Cyst Rupture	No (ref.)	–	1	–
	Yes	0.811	1.19	0.29–4.85
FIGO Stage	I–II (ref.)	–	1	–
	III	0.002	15.22	2.74–84.67

* HR (Hazard Ratio), risk ratio for death (n = 29 censored and n = 8 deaths)

CI, confidence interval; BMI, body mass index; Ref., reference category; FIGO, International Federation of Gynecology and Obstetrics

**Table 5:** Results of the multivariate Cox regression analysis for overall survival (n = 37)

**Variable**	**Categories**	**P Value**	H.R.*	**95% CI**
FIGO stage	I–II (ref.)	–	1	–
	III	0.002	15.22	2.74–84.67

* HR (Hazard Ratio), risk ratio for death (n = 29 censored and n = 8 deaths)

CI, confidence interval; Ref., reference category, FIGO, International Federation of Gynecology and Obstetrics

**Figure 2:**
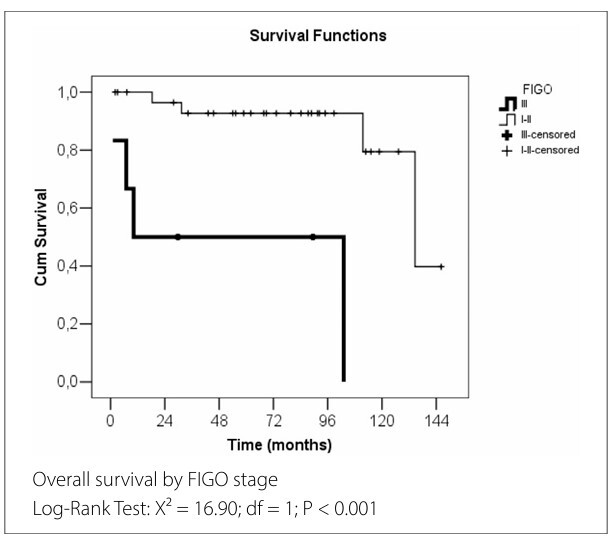
Overall survival analysis for significant variables (in months).

 Eight patients died during the study period (four with GBG 1 and four with GBG 2), and the mean overall survival for the entire cohort was 114.9 months. **Figure 2** presents the Kaplan–Meier overall survival curves for the variables identified as significant in **Tables 4** **and** **5**. 

**Figure 3:**
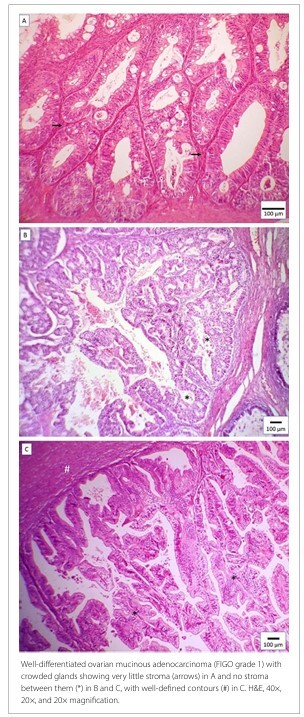
A, B, and C depict an expansile invasion pattern (GBG G1).

**Figure 4:**
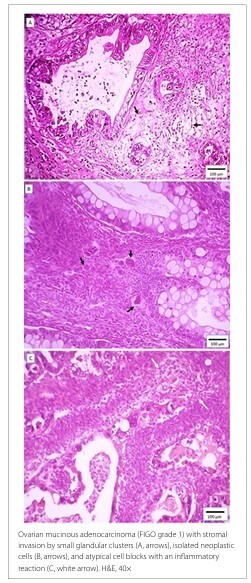
A, B, and C illustrate an infiltrative invasion pattern (GBG G1 or G2).

 Representative cases from this study are shown in **Figures 3** **and** **4**, illustrating ovarian mucinous carcinomas classified as GBG 1 (**Figure 3**) and with an infiltrative invasion pattern (**Figure 4**). 

## DISCUSSION

 Our study demonstrated that the GBG classification was correlated with disease-free survival, with GBG 2 tumors associated with a 5.4-fold higher risk of recurrence compared to GBG 1 tumors. This finding is consistent with the results reported by Momeni-Boroujeni et al.,^13^ who also observed a higher recurrence likelihood in GBG 2 tumors than in GBG 1 tumors. Notably, in contrast to GBG grading, the FIGO histological grading was not associated with recurrence risk in either study. 

 The prognostic role of FIGO histological grading remains controversial. A study involving women with tumors presumably confined to the ovary found that lymph node metastasis, although rare (1.4%), occurred more frequently in women with poorly differentiated tumors (histological grade 3).^15^ Because ovarian mucinous carcinoma is rare and typically diagnosed at an early stage, divergent results across studies are expected. The FIGO grading system is based on the proportion of solid growth and the presence of nuclear atypia, providing a morphological assessment of tumor differentiation.^9^ However, these features may not fully represent tumor biology. In contrast, GBG considers infiltrative invasion, a feature associated with greater aggressiveness, higher recurrence risk, and poorer prognosis. Therefore, GBG may offer a more accurate prognostic assessment by identifying invasive features that are not captured by morphology-based grading alone.^11,13^

 The National Comprehensive Cancer Network^®^ (NCCN) recommends that, in cases of ovarian mucinous carcinoma diagnosed during intraoperative frozen-section analysis, lymphadenectomy may be omitted if no suspicious lymph nodes are present.^3^ Additionally, the NCCN guidelines suggest that chemotherapy may or may not be prescribed in cases of tumor rupture in stage I expansile-type mucinous tumors.^3^ Supporting these recommendations, large-cohort studies have shown that systematic lymphadenectomy may be omitted in tumors that appear to be at stage I.^16,17^ However, these studies did not differentiate between expansile and infiltrative subtypes. In our study, complete staging with pelvic and para-aortic lymphadenectomy and tumor rupture were not associated with disease-free or overall survival. 

 Women with infiltrative subtype tumors are more often diagnosed at an advanced stage, have a higher incidence of lymph node metastasis, undergo more frequent restaging after initial surgical staging, and experience poorer oncologic outcomes than women with expansile subtype tumors.^4^ A recent study involving 409 women with stage I ovarian mucinous carcinoma found that complete staging with lymphadenectomy was associated with improved overall survival in the infiltrative subtype, but not in the expansile subtype.^18^ In addition, Algera et al.^8^ suggest that omitting peritoneal staging (peritoneal washing, peritoneal biopsies, and omentectomy) is likely safe for expansile tumors at stage I. The finding that GBG 2 tumors are associated with a higher recurrence risk provides a valuable threshold for defining the infiltrative component, enabling the classification of tumors into two groups with distinct prognostic implications. This distinction may support more tailored management strategies. 

 One important limitation of our study is the small number of recurrence and death events (three and eight, respectively), which restricts the statistical power of our analyses and could lead to overfitting of the Cox regression model. Therefore, survival outcomes should be interpreted with caution. Overall, our findings support incorporating invasion-based criteria, such as those used in the GBG system, into the pathological assessment of ovarian mucinous carcinoma. This new classification could guide surgical management, with systematic lymphadenectomy indicated for GBG 2 tumors and potentially omitted in GBG 1 cases. Adjuvant chemotherapy is currently recommended for patients with infiltrative-type mucinous ovarian carcinomas from stage Ib onwards.^3^ Incorporating the GBG classification may further refine this recommendation, with adjuvant chemotherapy potentially considered for GBG 2 tumors at these stages. Although further validation is needed, this approach could contribute to more tailored treatment decisions and also guide the development of post-treatment surveillance protocols, with closer follow-up recommended for patients with a poorer prognosis (GBG 2). 

## CONCLUSION

 While the present findings align with previously published data, they should be interpreted with caution, given the limited sample size and retrospective nature of the study. Additional validation using larger, independent cohorts is essential before any firm conclusions can be drawn. Nonetheless, the observed trends suggest that the GBG grading system may have potential value in refining risk assessments for mucinous ovarian carcinomas. 

## Data Availability

The data that support the findings of this study are available from the corresponding author, Adriana Yoshida, upon request.
